# Alveolar paratesticular rhabdomyosarcoma mimicing epididymitis: Case report and literature review: Erratum

**DOI:** 10.1097/MD.0000000000011674

**Published:** 2018-07-20

In the article, “Alveolar paratesticular rhabdomyosarcoma mimicing epididymitis: Case report and literature review”,^[1]^ which appeared in Volume 97, Issue 25 of *Medicine*, Dr. Jun Xin's correspondence address should appear as “Department of Urology The First Hospital of Quanzhou Affiliated to Fujian Medical University, 250 East Street, Licheng District, Quanzhou 362000, Fujian, China (e-mail: 15859516868@163.com).”

The hospital name in the affiliations should appear as “The First Hospital of Quanzhou Affiliated to Fujian Medical University.”
